# Scrotal perforation of peritoneal catheter: a rare complication of ventriculoperitoneal shunts

**Published:** 2012-11

**Authors:** Hamid Reza Saeidi Borojeni, Taravat Fakheri, Sepehr Saeidi Borojeni

**Affiliations:** ^*a*^Department of Neurosurgery, Kermanshah University of Medical Sciences, Kermanshah, Iran.; ^*b*^Kermanshah University of Medical Sciences, Kermanshah, Iran.; ^*c*^Tehran University of Medical Sciences, Tehran, Iran.

## Abstract

The standard treatment for the hydrocephalus is ventriculoperitoneal shunts (VPS) operation that diverts the excess accumulated cerebrospinal fluid (CSF) from the ventricles or other CSF containing spaces to another area. The VPS operation has been reportedly associated with risk factor for different organs due to peritoneal catheter migration including umbilicus, anus, vagina, mouth, intestine, internal jugular vein, chest, liver.

**Case:**

A 45-day-old male infant was admitted to emergency department of Imam Reza hospital (Kermanshah Iran) with a history of fever, restlessness, leakage of clear fluid through diaper from 2 days ago which exited; appearing from tip of the white tube extruding from scrotum. The infant was born with the myelomeningocele and paraplegia. Therefore, he had previously undergone a surgery at the age of 3 days. One week later the infant developed progressive hydrocephalus and a medium–pressure VP shunt was inserted. He was discharged 3 days later with good health condition and the sutures were removed 12 days later. After a month the infant was brought to the emergency department in ill condition with the symptoms described above. At the PE fever detected and wet diaper was seen and by shunt pump compression clear fluid emerged from the tip of catheter. The CSF analysis revealed meningitis, no growth of pathologic organism 48 hours post-culture. The patient was hospitalized and appropriate antibiotic treatment was started. 10 days later CSF became acellular and the shunt was removed and another VP shunt was inserted contralaterally. After 4 days scrotal orifice was cured and the infant discharged.

Previous stories have reported many VS shunt-associated complications such as mechanical failure, functional failure, infections, obstruction, disconnection, migration, hematoma, and slit ventricle syndrome. Mechanical failures consist of obstruction, fracture, disconnection, migration. A search performed in the PubMed showed that until 2006 there has been no report of any case of scrotal skin perforation. Removing the shunt or replacing it with another one can be a good option for the management of any VS shunt failure. In case of leaking fluid, proximal diversion is recommended, while following the treatment of any infection, a new shunt insertion in another site is recommended.

**Keywords:**

Hydrocephalus, Ventriculoperitoneal shunt, Infant, Scrotal perforation, Peritoneal catheter


					Volume 4, Suppl. 1 Nov 2012
Publisher: Kermanshah University of Medical Sciences
URL: http://www.jivresearch.org
Copyright:
Congress of Iranian Neurosurgeons, 14-16 November 2012, Kermanshah, Iran
Paper No. 80
Scrotal perforation of peritoneal catheter: a rare complication
of ventriculoperitoneal shunts
Hamid Reza Saeidi Borojeni a
, Taravat Fakheri a,*
, Sepehr Saeidi Borojeni c
a
Department of Neurosurgery, Kermanshah University of Medical Sciences, Kermanshah, Iran.
b
Kermanshah University of Medical Sciences, Kermanshah, Iran.
c
Tehran University of Medical Sciences, Tehran, Iran.
Abstract:
The standard treatment for the hydrocephalus is ventriculoperitoneal shunts (VPS) operation that diverts the excess
accumulated cerebrospinal fluid (CSF) from the ventricles or other CSF containing spaces to another area. The VPS
operation has been reportedly associated with risk factor for different organs due to peritoneal catheter migration
including umbilicus, anus, vagina, mouth, intestine, internal jugular vein, chest, liver.
Case: A 45-day-old male infant was admitted to emergency department of Imam Reza hospital (Kermanshah Iran)
with a history of fever, restlessness, leakage of clear fluid through diaper from 2 days ago which exited; appearing
from tip of the white tube extruding from scrotum. The infant was born with the myelomeningocele and paraplegia.
Therefore, he had previously undergone a surgery at the age of 3 days. One week later the infant developed
progressive hydrocephalus and a medium-pressure VP shunt was inserted. He was discharged 3 days later with
good health condition and the sutures were removed 12 days later. After a month the infant was brought to the
emergency department in ill condition with the symptoms described above. At the PE fever detected and wet diaper
was seen and by shunt pump compression clear fluid emerged from the tip of catheter. The CSF analysis revealed
meningitis, no growth of pathologic organism 48 hours post-culture. The patient was hospitalized and appropriate
antibiotic treatment was started. 10 days later CSF became acellular and the shunt was removed and another VP
shunt was inserted contralaterally. After 4 days scrotal orifice was cured and the infant discharged.
Previous stories have reported many VS shunt-associated complications such as mechanical failure, functional failure,
infections, obstruction, disconnection, migration, hematoma, and slit ventricle syndrome. Mechanical failures consist of
obstruction, fracture, disconnection, migration. A search performed in the PubMed showed that until 2006 there has
been no report of any case of scrotal skin perforation. Removing the shunt or replacing it with another one can be a
good option for the management of any VS shunt failure. In case of leaking fluid, proximal diversion is
recommended, while following the treatment of any infection, a new shunt insertion in another site is recommended.
Keywords:
Hydrocephalus, Ventriculoperitoneal shunt, Infant, Scrotal perforation, Peritoneal catheter
* Corresponding Author at:
Taravat Fakheri: Associated professor of Obs & Gyn Department, Maternity Research Center of Kermanshah University of Medical Sciences,
Kermanshah, Iran, Email: tfakheri@kums.ac.ir, (Fakheri T.).

				

